# Eggshell Based Nano-Engineered Hydroxyapatite and Poly(lactic) Acid Electrospun Fibers as Potential Tissue Scaffold

**DOI:** 10.1155/2019/6762575

**Published:** 2019-05-02

**Authors:** Vitus A. Apalangya, Vijaya K. Rangari, Boniface J. Tiimob, Shaik Jeelani, Temesgen Samuel

**Affiliations:** ^1^Department of Food Process Engineering, School of Engineering Sciences, University of Ghana, Legon, Accra, Ghana; ^2^Department of Material Sciences and Engineering, College of Engineering, Tuskegee University, Tuskegee, AL 36088, USA; ^3^Department of Pathobiology, College of Veterinary Medicine, Nursing and Allied Health, Tuskegee University, Tuskegee, AL 36088, USA

## Abstract

Nanocomposite electrospun fibers were fabricated from poly(lactic) acid (PLA) and needle-like hydroxyapatite nanoparticles made from eggshells. The X-ray diffraction spectrum and the scanning electron micrograph showed that the hydroxyapatite particles are highly crystalline and are needle-liked in shape with diameters between 10 and 20 nm and lengths ranging from 100 to 200 nm. The microstructural, thermal, and mechanical properties of the electrospun fibers were characterized using scanning electron microscope (SEM), thermogravimetric analysis (TGA), dynamic scanning calorimetry (DSC), and tensile testing techniques. The SEM study showed that both pristine and PLA/EnHA fibers surfaces exhibited numerous pores and rough edges suitable for cell attachment. The presence of the rod-liked EnHA particles was found to increase thermal and mechanical properties of PLA fibers relative to pristine PLA fibers. The confocal optical images showed that osteoblast cells were found to attach on dense pristine PLA and PLA/HA-10 wt% fibers after 48 hours of incubation. The stained confocal optical images indicated the secretion of cytoplasmic extension linking adjoining nuclei after 96 hours of incubation. These findings showed that eggshell based nanohydroxyapatite and poly(lactic acid) fibers could be potential scaffold for tissue regeneration.

## 1. Introduction

Hydroxyapatite (HA), like other calcium phosphate bioceramics, has demonstrated immense bone integration and ingrowth capabilities [[1–6]. HA is nontoxic and biodegradable and easily adsorbs onto surfaces of bioactive molecules. These characteristics make it useful for both tissue engineering (TE) and drug delivery applications [2, 7]. However due to the inherent brittle nature of hydroxyapatite nanoparticles, they are usually incorporated into polymeric nanocomposites to ensure easy processing [8, 9]. Thus, the elastic polymeric matrix overcomes the intrinsic brittleness of the bioceramic by improving upon its design flexibility. The HA nanoparticles play a dual function as they provide the scaffolds with bioactive bone forming material while tailoring their high stiffness to forming strong cell and tissue supports [10, 11]. Nanoparticles particularly HA nanoparticles or rods can be excellent fillers of damage and bone defects as their size and surface morphology match those of natural bones [12]. Moreover the porous surfaces of fibers are amenable to carrying bioactive and growth factors which promote speedy tissue formation and integration when they are incorporated with HA nanoparticles [13].

Polymer/HA scaffolds have been produced using different varieties of technologies such as extrusion, stereo lithography, coprecipitation, electrospinning, etc. However, the simplicity of experimental setup, low cost, the high porosity, and high interconnectivity of electrospun fibers makes them ideal tissue scaffolds [14–17]. HA nanoparticles are incorporated into the polymer matrix either through biomineralization of pristine polymer fiber mats or coelectrospinning of the polymer and the HA nanoparticles [4, 18]. The incorporation of most calcium phosphate ceramics into polymer nanocomposite electrospun fibers involves coelectrospinning of polymer containing calcium and phosphate precursors. Current reports on the effect of hydroxyapatite nanoparticles on the mechanical properties of polymer composites seem to produce mixed results as the nanomaterial appeared to enhance the mechanical properties of HA/PLA electrospun composites in some studies, yet in others, the mechanical properties are deteriorating. Abdal-hay et al. [19] have showed that the improvement in the mechanical properties of N6 electrospun fibers was due to good dispersion and alignment of plate-like hydroxyapatite nanoparticles on the surface of the N6 fibers. The deterioration of mechanical properties could be a function of the poor miscibility of the inorganic HA nanoparticles and the polymer matrix due to the inherent inorganic-organic phase separation and the concomitant agglomeration of the HA nanoparticles. Furthermore the shape of the HA particles can play a key role as the shape of the particles are related to the aspect ratio. High aspect ratio HA nanoparticles are more likely to contact and interact with more neighboring polymer matrix than spherical HA nanoparticles during electrospinning when they are oriented along the length of the fiber. This interaction could lead to stress transfer from the polymer matrix to the HA nanoparticles resulting in an increase in mechanical properties [20]. Moreover high aspect ratio HA particles if well align on the surface of the fibers could ensure sufficient and uniform distribution of HA nanoparticles on the surface of the fibers which may lead to uniform cell growth at low loading of the HA nanoparticles. Hydroxyapatite synthesized from biobased materials such as eggshells has been suggested to exhibit interesting potential tissue support capabilities due to the likely high biocompatibility associated with this precursor biomaterial [21–23].

The objective of this study is to investigate how needle-liked or rod-liked hydroxyapatite nanoparticles made from eggshells affect the mechanical, thermal, and morphological as well as tissue supporting potential of poly(lactic) acid electrospun fibers.

## 2. Materials and Methods

Raw white eggshells were provided by American Dehydrated Foods, Atlanta, GA. Ethanol (99.5% purity, absolute), nitric acid (65% HNO_3_), ammonium hydroxide (28% NH_4_OH), semiconductor grade phosphoric acid (85% H_3_PO_4_), poly(lactic acid) (PLA, Mw: 76000), anhydrous chloroform (≥99% purity), and dimethyl sulfoxide (≥99% DMSO) were purchased from Sigma-Aldrich Chemical Company St. Louis, MO. Human osteoblast cells (ATCC CRL 11372) were purchased from American Type Culture Collection (ATCC); Dulbecco's modified eagle medium (DMEM) was purchased from Fisher scientific. Penicillin and streptomycin were purchased from Lonza. Fetal bovine serum (FBS Lot no. 8SB013) was purchased from VWR. Hematoxylin and eosin reagents were purchased from Sigma-Aldrich.

### 2.1. Synthesis of Egg Shell Nanohydroxyapatite (EnHA)

The eggshell calcium carbonate intermediate precursor reagents were prepared according to previously reported method except that two balls of different diameters were used in the milling process [24, 25] and also described elsewhere by Apalangya et al., but briefly described here as follows. The dried pulverized eggshells were sieved using stainless-steel sieves. Four grams of the eggshells was uniformly dispersed in ethanol and water (1:1v/v) mixture. The eggshell suspension was transferred to a stainless-steel canister and fixed in a Spex Sample Prep Mixer/Mill 8000. The suspension was milled for 3 hours in a 10 ml polypropylene glycol using two sets of 6 of 3 mm and 12 of 6 mm diameter stainless-steel balls. The milled eggshells were cooled down and washed using water and ethanol mixture. The product was dried at 40°C for 3 hours in the oven to ensure complete removal of ethanol. The synthesis was carried out according to same previously reported method except that the temperature of the CEM microwave was kept at 50°C and reaction lasted for 15 minutes and briefly described as follows [26]. A diammonium hydrogen phosphate (NH_4_)_2_HPO_4_ (0.0036 M) was added dropwise while under stirring for 15 min and the pH made up to 11 using ammonium hydroxide solution. The resulting mixture was then transferred to a CEU microwave reaction vessel. The reaction mixture was irradiated for 30 minutes at 50°C and purged with argon gas at a pressure of 60 psi. The crystals were collected and washed thoroughly using a mixture of ethanol and deionized water (1:1 v/v) and then centrifuged at 12,000 rpm for 10 min to remove the solvent. The washing was repeated two more times and as-synthesized eggshell nanohydroxyapatite (EnHA) particles were vacuum-dried overnight and sample was collected in a desiccator ready for characterization.

#### 2.1.1. Transmission Electron Microscopy (TEM)

The size, shape of the nanoparticles, and the morphology of the electrospun fibers were analyzed using a JEOL-2010 transmission electron microscope (TEM), operating at 80 kV. Samples were prepared by uniformly dispersing 5.0 mg EnHA nanoparticles sample in 10 ml of ethanol using sonication bath. The colloidal solution was dropped on a carbon grid (carbon coated copper grid) and dried at room temperature.

#### 2.1.2. X-Ray Diffraction (XRD)

The crystallinity of the synthesized EnHA nanoparticles was investigated by X-ray Rigaku DMAX 2100 diffractometer with monochromatic CuK *α* radiation (*λ* = 0.154056 nm) generated at 40 kV and 30 mA. The intensity data were collected over the 2*θ* range 10-80° at a scan rate of 0.5° 2*θ* per minute. The pattern peaks from the diffraction spectra were analyzed by indexing with known compounds in Jade 9 software.

### 2.2. Preparation of PLA Electrospun Fibers

Polymer solutions were prepared by dissolving 1.0 g of poly(lactic acid) (PLA, Mw: 76000) (Sigma-Aldrich Chemical Company St. Louis, MO) in anhydrous chloroform (≥99% purity) (Sigma-Aldrich Company) (St. Louis, MO) in an Erlenmeyer flask. The flask and its contents were heated at 50°C for 30 minutes while being stirred during which all polymer granules dissolved and dispersed uniformly. The EnHA nanoparticles weighing 2.5, 5, and 10 wt% based on the weight of PLA which correspond to 0.025, 0.05, and 0.1 g were uniformly dispersed in 10 ml of chloroform and stirred on a magnetic plate. The mixture was sonicated in a sonication bath for 15 minutes during which all the EnHA nanoparticles dispersed uniformly. The EnHA solution is then added to the polymer solution drop wise and stirred on a magnetic plate for 30 minutes at 40°C until a homogenous mixture was obtained. The PLA and PLA/EnHA suspensions were loaded in a 5-ml plastic syringe with a stainless-steel needle (with internal diameter = 0.584 mm) positioned in the pump for the electrospinning process. A high voltage power supply was connected to the needle tip and the positive DC voltage set to 12.5 kV. The flow rate of the suspension was controlled and maintained at 1.5 mlh^−1^ by a Harvard 100 syringe pump. A grounded aluminum foil wrapped around a wooden rectangular board was placed at 20 cm from the needle tip as the collector. The electrospun fibers were collected as nonwoven mats (random mesh) on the collector. All fibers were vacuum-dried at 60°C overnight and then stored in a desiccator prior to characterization.

### 2.3. Scanning Electron Microscope (SEM) of Fibers

The morphology and size of the as-synthesized EnHA nanoparticles and the electrospun fibers were investigated using JEOL JSM 5800 Scanning Electron Microscope. In addition, the diameters of the electrospun fibers were also determined using this technique. The hydroxyapatite powder samples were spread thinly on a double sided adhesive conductive carbon tape. The samples were sputter coated with layer of gold/palladium conductive particle using a sputter coater Hummer 6.2 with the aim to prevent surface charging of the sample due to electron build up resulting from the absorption of the electrons by the nonconductive sample.

### 2.4. Thermal Analysis

The decomposition profile of the pristine PLA and the PLA/EnHA electrospun fibers was determined by subjecting the fibers to heating at room temperature to 1000°C in nitrogen environment. The measurements were carried out in Mettler Toledo TGA/SDTA 851e apparatus. The weight of the PLA/EnHA fibers (about 13.5g) was measured into aluminum oxide pans. The temperature program was set at 25 – 1000°C and the heating rate at 5°C/min with nitrogen gas flow 40 psi.

Differential scanning calorimetry (DSC) measurements of the PLA and PLA/EnHA mats were performed with a Mettler Toledo DSC/SDTA 851e apparatus. To remove any thermal history, samples of 5–10 mg were heated to 200°C at a heating rate of 30°C min^−1^, and maintained at this temperature for 5 min followed by cooling to room temperature at a rate of 10°C min^−1^. During subsequent test runs, the samples were reheated to 200°C at a heating rate of 10°C mi^−1^ and test data collected. The crystallinity of the samples was calculated using the following equation:

(1) $$\begin{array}{c} X_{c}=\frac{{∆H}_{m}-{∆H}_{cc}}{{∆H}_{m0}}\;\;x\;\;100 \end{array}$$

where Xc (%) is the crystallinity, ∆*Hm* (J/g) is the heat of fusion from the second heating circle, ∆H_cc_ (J/g) is the heat of cold crystallization, and ∆H_m0_ is the heat of fusion for 100% crystalline PLA, taken as 93 J/g [27, 28]. The absolute crystallinity of PLA in the composites was calculated as Xp:

(2) $$\begin{array}{c} X_{p}=\frac{X_{c}}{w} \end{array}$$

where w is the weight fraction of PLA in the composites.

### 2.5. Tensile Testing of Fibers

Tensile properties of nanofibrous mats were determined at normal room temperature using a universal testing machine (Zwick Roell Z 2.5) at a crosshead speed of 50 mm/min. Samples were cut into 10 mm × 60 mm rectangular specimens from the electrospun membrane of 20–30 *μ*m thickness and used for mechanical studies. The ends of the rectangular specimens were supported on a paper grips and mounted vertically on mechanical gripping units of the tensile tester and a load of 20 N was applied for tensile measurements. A minimum of 5 tests were performed.

### 2.6. Cell Adhesion Studies

The fiber mats of pristine PLA and the PLA/10wt%EnHA were cut into circular discs with uniform thickness using a paper perforator. Before perforation, two pieces of polypropylene plastic were used to sandwich the dense fibers to ease handling and storage. Prior to use the circular disc samples (fibers) were removed from the desiccator and sterilized with UV radiation for 30 min. The samples were made hydrophilic by dipping them into 70% ethanol for 10 minutes followed by washing in sterilized PBS to remove the ethanol. Finally, discs were rinsed in the cell culture medium prior to use. The pristine PLA and PLA/5 wt% EnHA fiber discs were each placed in two 24-well plates. To each well containing fiber disc, the human osteoblastic cells (ATCC CRL 11372) were added in 96-well cell culture plates at a density of 2 × 10^4^ cells per well in Dulbecco's modified eagle medium (DMEM F12 1:1) supplemented with 10% fetal bovine serum, 100 units mL^−1^ penicillin, and 100 *μ*g mL^−1^ streptomycin. One set of plates (one for pristine PLA and one for PLA/10 wt% EnHA) were cultured at 37°C in 5% CO_2_ for 72 hours in 150 *μ*L DMEM culture medium. The medium was monitored every day for color change from pink to yellow for dense cell growth or for undesirable contamination. Clear yellowish medium was aspirated off and replaced with fresh media. At the end of the experiment, the medium was aspirated off and well rinsed three times with PBS. The discs were fixed with 4% formaldehyde solution for 10 minutes and washed three times with PBS. The fixed discs removed analysis by optical light microscopy (Olympus IX1). Another set of fibers with the same number of cells were allowed to incubate for 120 hours after which they appeared dense and incapable of been observed directly by optical microscopy. In order to observe if there is any other growth apart from cell attachment on fibers, the medium was aspirated off and washed three times with PBS. The fixed discs were removed and stained with hematoxylin and eosin reagents and paraffinized for microscopical analysis.

## 3. Results and Discussion

### 3.1. X-Ray Diffraction of Eggshell Nanohydroxyapatite (EnHA) Nanoparticles

**Figure 1:**
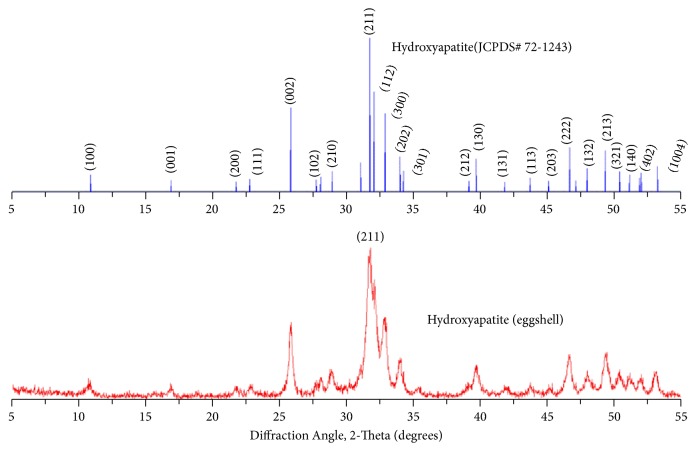
X-ray diffraction results of eggshell nanohydroxyapatite nanoparticles synthesized from eggshell compared with hydroxyapatite (JCPDS number 72-1245).

Depicted in Figure 1 is the XRD pattern of Egg nanohydroxyapatite (EnHA) nanoparticles made from eggshells source calcium carbonate with clearly defined and highly crystalline hexagonal structure with no evidence of any secondary phase formation. The sample peaks indicated a perfect match of the synthesized EnHA with the JCPDF card number 72-1243.

#### 3.1.1. Scanning Electron Microscope (SEM) of Eggshell Nanohydroxyapatite (EnHA) Nanoparticles

**Figure 2:**
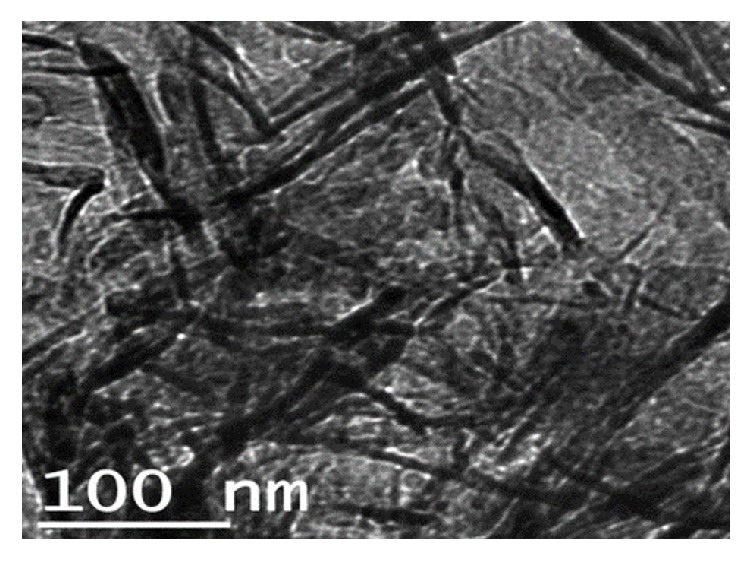
TEM micrographs of hydroxyapatite nanoparticles synthesized from eggshells.

The synthesis yielded noticeable nanohydroxyapatite particles with needle-liked shapes. The particles sizes are lying between 100 and 120 nm and their diameters range from 10 to 20 nm (Figure 2). These dimensions of the hydroxyapatite nanoparticles present large surface area and enable better biological responses when being in contact with osteoblastic cells [29].

#### 3.1.2. Morphologies Electrospun Fibers

**Figure 3:**
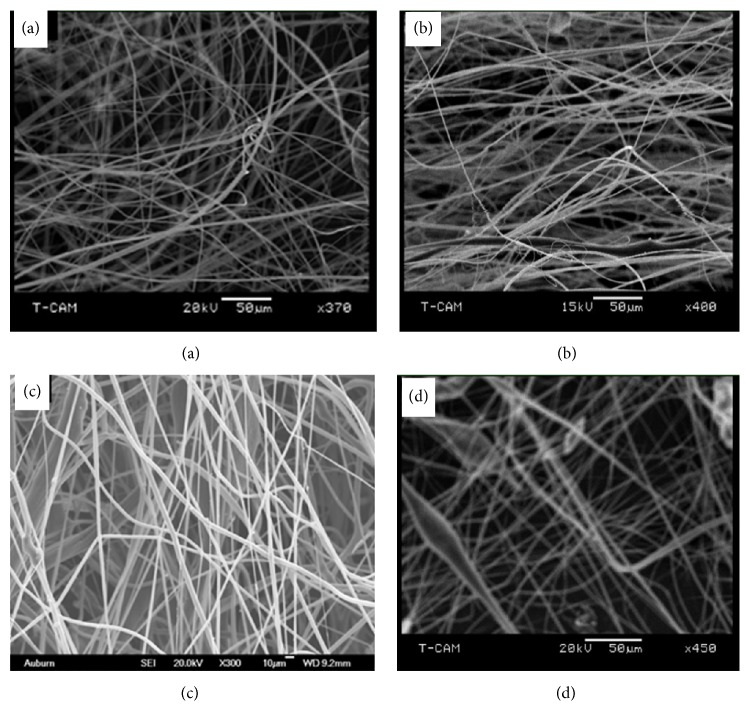
Low magnification SEM images of (a) pristine PLA, (b) PLA/EnHA, (c) PLA/2.5wt% EnHA5wt%, and (d) PLA/EnHAwt% electrospun fibers.

**Figure 4:**
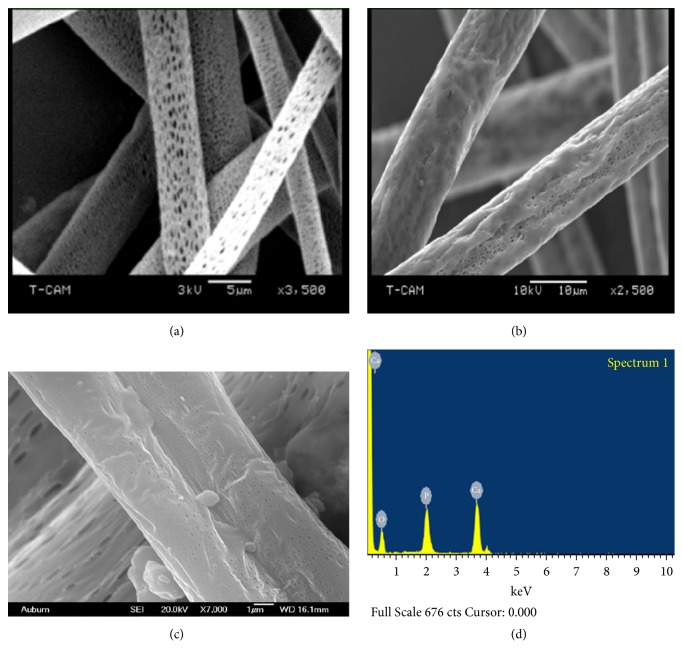
High magnification SEM micrographs of (a) neat PLA, (b) PLA/ 5 wt% EnHA, (c) PLA/10wt% EnHA, and (d) EDS spectrum of PLA/10 wt% EnHA.

SEM low magnification micrographs of pristine PLA and PLA/EnHA composite electrospun fibrous mats are shown in Figure 3. Generally all fibers exhibited uniform and homogenous diameters of approximately 5 *μ*m with the exception of the PLA/10 wt% EnHA fibers whose fiber diameters are uniform. As illustrated in Figure 4, the high magnification micrograph of the pristine PLA fibers showed that their surface microstructure is replete with numerous pores which are distributed uniformly on the surface of the pristine fibers. Even though there are also pores on the surface of the PLA/EnHA composite fibers, they occurred to a less extend as compared to the pristine fibers. The numerous appearance of the pores on the surface of the fibers in this study may be due to the volatilization of the solvent used in the fabrication process as well as the exothermic reaction within the polymers [28, 30]. In case of the PLA/EnHA fiber mats, it could be attributed to the solution immiscibility of the PLA polymers and EnHA nanoparticles; this probably caused phase separation between the polymer and the EnHA particles in solution resulting in the formation of pores on the surface of the fibers.

Additionally there was aggregation of particles on the surface of the PLA/10 wt% EnHA (as illustrated in Figure 4(c)) compared to the PLA/5 wt% EnHA composite fibers as shown in Figure 4(b). This likely reduced the visibility of the pores on the surface of the PLA/10 wt% EnHA fibers leaving the surface with the appearance of rough edges. In contrast the PLA/5 wt% EnHA fibers appeared cylindrical with uniformly distributed diameters. This aggregate of materials on the surface of the fibers especially more visible on the PLA/10 wt% EnHA fibers could be due to the aggregation of the hydroxyapatite nanoparticles or PLA polymer itself on the spinneret head which interfered with the continuous and uniform deposition of the fibers during the electrospinning process. In order to verify if the deposits were in fact EnHA, an EDS spectrum of the surface of the PLA/10 wt% EnHA fibers was performed. As shown in Figure 4(d) the presence of elemental Ca, P, and O on the surface of the PLA/EnHA 10 wt% fibers confirmed that the rough edges on the composite fibers were as a result of the presence of the hydroxyapatite.

These porous surfaces or rough edges could be desirable for cell attachment and could promote exchange or transport of liquids when these fibrous membranes are used as tissue supports [31–34].

#### 3.1.3. Thermal Analysis

**Figure 5:**
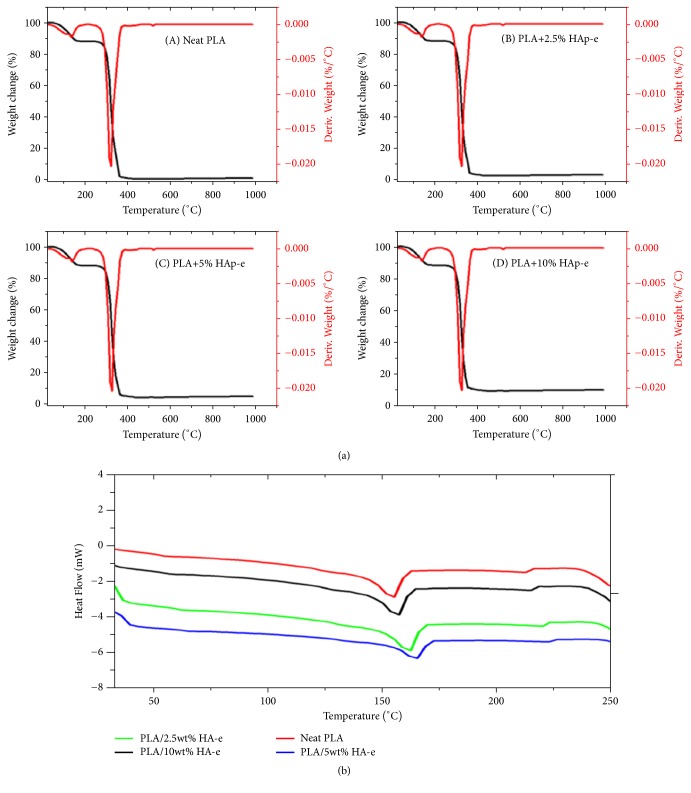
TGA (a) and (b) DSC curves of the electrospun neat PLA and PLA/EnHA fibers.

Figure 5(a) shows the thermogravimetric Analysis (TGA) profiles for the pristine PLA and the composite PLA/EnHA electrospun fibers. Generally, the increase loading of EnHA in the PLA/ EnHA fibers led to an improvement in the thermal decomposition temperature of the PLA/EnHA fibers relative to the pristine PLA fibers. Specifically, the percentage improvement in the decomposition temperature of PLA/EnHA fibers was as follows: 1.2%, 5.7%, and 1.4% corresponding to the PLA/2.5 wt% EnHA, PLA/5 wt% EnHA, and PLA/10 wt% EnHA, respectively, relative to the pristine PLA fibers. The significant increase in the decomposition temperature of the PLA/5 wt% EnHA fibers is an indication that this class of fibers exhibited superior heat resistance relative to the other fibers. As shown also in Table 1, the char yield increases with increase in the loading of the EnHA and in fact the char yield is reasonably closed to the amount of EnHA used in the initial formulation of the fibers. Since the amount of char is related to decrease in the release of volatiles or combustible gases, the increase inclusion of hydroxyapatite in the fibers increases the potential of the fibers as effective fire retardants and reduces the thermal conductivity of the fibers [35, 36]. Moreover, the increase in crystallinity as a function of increase in EnHA loading in the PLA fibers could result in the improvement in the heat resistance of the composite PLA/EnHA fibers.

While there is improvement in heat decomposition temperature of the PLA fibers with low incorporation of EnHA particles as shown in Figures 5(a) and 5(b), there was decline in decomposition temperature when the EnHA content was increased to 10 wt% relative to the pristine fibers. This could be due to aggregation of the EnHA particles. The increase of EnHA nanoparticles decreases the distance between individual EnHA nanoparticles and increases the possibility of interaction of the EnHA particles among themselves in solution. This interaction might have led to the formation of aggregates which weakened the strength of the fibers. The DSC curves of the neat and the PLA/EnHA electrospun fibers are shown in Figure 5(b) and summarized in Table 2. The glass transition temperatures (Tg) were obtained from the DSC curves as the inflection points of the heat flow curves [37]. The pristine PLA and PLA/EnHA electrospun fibers undergo three thermal changes as the samples are subjected to heating from 30 to 250°C. All samples showed three endothermic peaks which can be attributed to Tg and two melting temperatures, Tm.

The Tg of the neat PLA polymer is 60.1°C. This correlates with Tg literature values of PLA [38, 39]. The incorporation of 2.5 wt% of EnHA in the PLA led to a slight increase of 1.2°C in Tg relative to the pristine fibers. The PLA/5 wt% EnHA fibers showed a significant improvement in Tg by 5.7°C for PLA/5 wt% EnHA fibers.

However, there was virtually no improvement in the Tg for PLA/10 wt% EnHA fibers relative to the pristine PLA electrospun fibers. These improvements in Tg constituted approximately 1.1%, 5.7% and 0.9% corresponding to PLA/2.5 wt% EnHA, PLA/5 wt% EnHA, and PLA/10 wt% EnHA, respectively, relative to the neat PLA fibers. It is possible that the 2.5 wt% EnHA was not enough to restrict the PLA molecular chains but the 5 wt% EnHA was the critical concentration needed to restrict PLA molecular chains resulting in a higher glass transition temperature relative to the pristine fibers. However, as the HA content increased to 10 wt% of EnHA, the glass transition temperature of the PLA/10 wt% EnHA fibers decreased due to aggregation of the HA nanoparticles.

#### 3.1.4. Mechanical Properties of PLA and PLA/EnHA Electrospun Fiber Mats

To understand the influence of blending various percentage amount of EnHA nanoparticles on the mechanical properties of PLA/EnHA fibers, tensile testing was employed. The characteristic properties, maximum tensile strength, Young modulus, and elongation at break of the pristine PLA and PLA/EnHA fibers, are extracted from the load deformations curves and summarized in Table 2. There is a general improvement in mechanical properties (strength and modulus) as the percentage loading of EnHA in the PLA/EnHA fibers is increased. Particularly at loading range of EnHA between 2.5 and 5 wt%, the tensile strength of the PLA/2.5 wt% EnHA and PLA/5 wt% EnHA fiber mats relative to the tensile strength of the pristine fibers increased by 3- and 5-fold, respectively.

The Young modulus of the PLA/5 wt% EnHA fiber mats increased 20 times relative to the pristine fibers. This marked improvement of the tensile strength and the Young modulus is due to the increased in the stiffness of the fibers as evident by the decrease in percentage elongation yield. However, when the content of the EnHA nanoparticles was increased to 10%, the yield stress only increased slightly relative to the neat but marked a significant decreased in yield stress in comparison to the PLA/5 wt% EnHA fiber mats. This degradation of properties may be due to the aggregation of the HA nanoparticles at high loading of EnHA particles. This reduces the interaction of fibers with each other. Particularly, the deposition of EnHA particles on the surface of PLA/10 wt% EnHA as shown in Figure 4(c) likely reduced the attraction between fibers. This led to reduction in crystallinity as shown in Table 1 which suggested strongly that high loading resulted in the reduction of the cohesive forces between fibers leading to degrading mechanical properties. However, some studies have indicated that hydroxyapatite nanoparticles turn to degrade the mechanical properties of hydroxyapatite polymer composite [40]. The increase in tensile stress and Young modulus at low loading of EnHA nanoparticles may be due to the high alignment or orientation of the needled-liked particles on the PLA fiber mats. This is consistent with previous reports which showed that alignment of nanoplatelet hydroxyapatite on nylon electrospun fibers turns to improve their mechanical properties [17]. The good alignment of the EnHA particles on the surface of the fibers turns to promote good interfacial hydrogen bonding between the EnHA particles and the PLA polymer causing a good stress transfer from the matrix to the nanofibers [26]. Another possible reason is the micromechanical interlocking, which is likely to be induced between the EnHA nanoplatelets and the fiber surface molecules in composites.

#### 3.1.5. Fibroblast Cells Adhesion Studies

**Figure 6:**
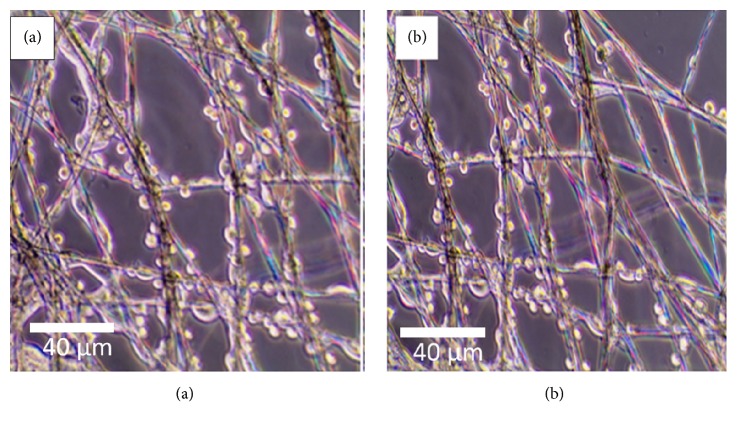
Optical microscope images of ATCC CRL 11372 osteoblast cells adherent to (a) PLA and (b) PLA/5 wt% EnHA.

Figures 6(a) and 6(b) depict the attachment and adhesion of cells to pristine PLA and PLA/5 wt% EnHA, respectively, after 48 hours of incubation. It is noticeable from both images that the fibers served as continuous support for attachment and growth of the fibroblast cells. This marked an important step in developing and generating biological tissues as tissue implants. The creation of sufficiently bioactive, rough, and hydrophilic fiber surfaces promoted the attachment and differentiation of cells on fiber mats.

It is evident that the loading of the hydroxyapatite particles successfully produced PLA fibers with suitable mechanical properties and bioactive surface material which is familiar to the cells as the cells could be seen clinging to the surface of individual fibers after few days of incubation [41]. After 48 hours of incubation there were no clear differences in attachment and growth between the pristine fibers and the PLA/EnHA fibers. This may be due to the fact that other factors such as surface hydrophilicity and roughness may be more critical to cell attachment and growth at the early days of cell incubation. However, as the cells grow and differentiate into tissues, the surface presence of hydroxyapatite may now play a prominent role. It is evident from the SEM images that both the pristine PLA and PLA/5 wt% EnHA fibers exhibited some degree of roughness and have been treated in similar fashion to induce hydrophilicity before seeding of the cells. The advantage of using the optical microscope to follow the adherence of the cells on the fibers is important as it enables good view of the attachment and growth of individual cells on the surface of the fibers

**Figure 7:**
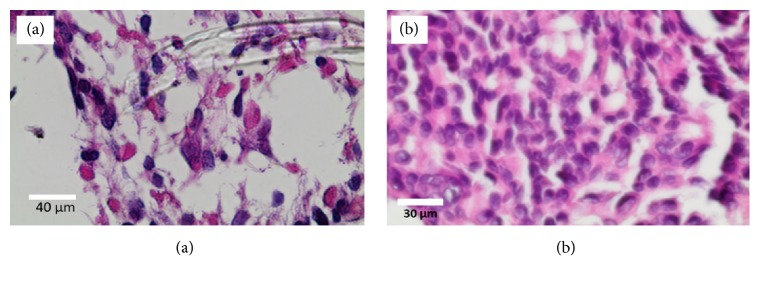
Optical microscopic images of histological stained section of human osteoblast cells grown on (a) pristine PLA fibers and (b) PLA/10 wt% HA fibers.

To assess and visualize how cells are interacting with neighboring cells, staining similar to immunochemistry staining analysis was performed where the cells were fixed to maintain their morphology, distinctively stained, and imaged using optical microscope. As illustrated in Figure 7, the histological processing of the paraffin blocks fibers appeared dissolved leaving trails around which stained cells are visible (purple) on hydroxyapatite modified PLA fibers (PLA/5 wt% EnHA). The nuclei of the cells appeared deep purple with light purple surroundings. These lighter purple extensions appeared as cytoplasmic connections that stretch out to neighboring cells from adjacent cells. These cell secretions marked the beginning of the joining of neighboring cells to form a tissue. This is consistent with earlier study that showed that there was excellent cell attachment and spreading on the surface of ACP-PLA and HA-PLA composite nanofibers with accompanying appearance of cell cytoplasmic extensions typical of osteoblast cells [42]. Optical imaging unlike advance electronic imaging techniques such as scanning electron microscopy does not require specialized sample treatments and therefore allows for easy following of the early days of individual cell attachment and growth to forming tissues on the scaffold. The presence of HA nanoparticles on surface of the PLA/HA fibers might have played a role of enhancing interactions with preosteoblasts, directed cell anchorage and movement, and further regulated cell differentiation and matrix syntheses. In addition, the presence of these HA nanoparticles may stimulate the formation of similarly structured but nonstoichiometric nanostructured hydroxyapatite, which are a mixture of amorphous calcium phosphate and crystalline hydroxyapatite nanomaterials. Amorphous calcium phosphate material can be beneficial in enhancing more tissue formation than hydroxyapatite since the poorly crystalline and amorphous calcium phosphates (ACP) are known to exhibit faster resorption characteristics. The resorption of ACP would release calcium and phosphate ions into the surrounding environment, which will be responsible for the osteoconductive properties.

## 4. Conclusion

Hydroxyapatite (EnHA) prepared from eggshells was successfully coelectrospun with poly(lactic acid) (PLA) to form PLA/EnHA fibers. The composite fibers exhibited improved thermal and mechanical properties relative to the pristine PLA electrospun fibers at lower loading of EnHA; especially the maximum tensile stress was obtained in composite fibers with 5 wt% EnHA content. Osteoblast cells successfully adhered and grew on the PLA/EnHA fibers. The immunohistochemistry staining showed that the cells could grow on the fibers to confluence and secrete components of the extracellular matrix. However more work will need to be done to identify the nature of the cell secretions.

## Figures and Tables

**Table 1:** TGA results for neat PLA and PLA/EnHA electrospun fibers.

Samples	Decomposition Temperature (°C)	Residue (g)	Tg (°C)	Tm (°C)	∆*Hm* (J/g)	∆*Hcc* (J/g)	Xc (%)	Xp (%)
Neat PLA	350.8	0.2	60.1	164.5	36.9	36.8	0.0	0.00
PLA/ 2.5wt% EnHA	353.6	1.9	60.3	165.7	33.7	31.8	2.04	2.26
PLA/5 wt% EnHA	357.5	4.2	62.7	167.1	30.5	23.9	7.10	7.55
PLA/10 wt% EnHA	354.5	6.3	60.4	164.1	25.8	23.6	2.4	2.63

**Table 2:** Tensile properties of PLA/EnHA fibers.

Samples	Maximum Tensile Stress (MPa)	Young's Modulus (MPA)	Elongation at Break (%)
PLA	1.15	3.8	18.50
PLA/2.5 wt% EnHA	3.65	39.4	9.50
PLA/5 wt% EnHA	5.40	72.2	7.70
PLA/10 wt% EnHA	2.3	39.3	5.00

## Data Availability

The data used to support the findings of this study are available from the corresponding author upon request.
